# Antianhedonic and Antidepressant Effects of Affron^®^, a Standardized Saffron (*Crocus Sativus* L.) Extract

**DOI:** 10.3390/molecules25143207

**Published:** 2020-07-15

**Authors:** Laura Orio, Francisco Alen, Antonio Ballesta, Raquel Martin, Raquel Gomez de Heras

**Affiliations:** Department of Psychobiology and Behavioral Sciences Methods, Faculty of Psychology, Complutense University of Madrid, 28223 Madrid, Spain; lorio@psi.ucm.es (L.O.); aj.ballesta@ucm.es (A.B.); rmarti14@ucm.es (R.M.); rgomezhe@psi.ucm.es (R.G.d.H.)

**Keywords:** saffron, affron^®^, depression, anxiety, antioxidant

## Abstract

Anxiety and depression have high prevalence in the general population, affecting millions of people worldwide, but there is still a need for effective and safe treatments. Nutritional supplements have recently received a lot of attention, particularly saffron. Thus, several pre-clinical studies support a beneficial role for bioactive compounds, such as saffron, in anxiety and depression. Here we used an animal model of depression based on social isolation to assess the effects of affron^®^, a standardized saffron extract containing ≥3.5% of total bioactive compounds safranal and crocin isomers. Affron^®^ was administered both through the oral and the intraperitoneal routes, and several tasks related to anxiety and depression, such as the elevated plus maze, the forced swimming test or the sucrose preference test, were assessed. These tasks model key features of depressive states and anxious states relating to fear, behavioral despair or anhedonia, the lack of motivation and/or pleasure from everyday activities, respectively. Animals receiving oral affron^®^ displayed behaviors congruent with improvements in their anxious/depressive state, showing the enhanced consumption of a sweet solution, as well as an increase in certain escape responses in the forced swimming test. Our data support a beneficial role for oral saffron in anxious/depressive states.

## 1. Introduction

Anxiety and depression are widely acknowledged as psychiatric disorders of global concern that could compromise human welfare [1], thus, the two conditions often co-exist; between 40% and 60% of patients with a common mental health disorder meet criteria for both anxiety and depression [2,3]. According to the World Health Organization data, there is high prevalence for depression and anxiety, affecting more than 300 million people worldwide, and they include the mixed depressive and anxiety disorder in their International Classification of Diseases [4]. These two conditions share common risk factors and many symptoms that can be regarded as existing on a spectrum of the disorder [5].

Mood alterations, including clinical depression, range from non-clinical low mood to major depression [6,7,8]. This low mood can include many of the symptoms characteristic of depression, such as sadness, crying, fatigue, pessimism, changes in appetite, changes in sleep patterns and anhedonia [6,7,9]. Currently there is no pharmacological treatment for low mood, and prescription medications are not only deemed inappropriate but also ineffective [10,11].

The conventional management of depression and anxiety disorders includes cognitive behavioral therapy, pharmacotherapy or electroconvulsive therapy [8,12]. However, despite the availability of numerous classes of drugs for the treatment, full remission of disease symptoms has remained elusive. Nevertheless, the clinical use of these drugs is limited by their characteristic side effects and poor tolerability profile [13]. Several natural compounds are being considered for their possible role in the treatment of mood disorders, including saffron, St John’s wort, tryptophan and omega-3 fatty acids, among others [14,15,16].

Saffron dried stigmas from *Crocus sativus* L. are conventionally used as a spice, textile dye or even as a perfume, due to its organoleptic properties. In addition, it is also widely known in traditional medicine for eye problems, headaches, genitourinary complications and other illnesses in different cultures [17,18,19,20]. The quality of saffron is determined by its secondary metabolites, such as picrocrocin, which is responsible for the bitter taste, safranal, which is related to saffron aroma, and crocins, which provide the color [21,22,23]. These compounds, mainly safranal and crocin isomers, as well as their metabolic derivate, crocetin, are related to antioxidant [24,25], anxiolytic [26,27], neuroprotective [28], anti-inflammatory [29,30,31] antidepressant [32,33,34] and anti-Alzheimer properties, which have been proven in several clinical trials [35].

As with most psychiatric disorders, the etiopathology of depression appears to be complex and multifactorial, including genetic, social and mood regulation mechanisms, among others. Alterations in neurotransmitter levels, including the abnormal regulation of cholinergic, catecholaminergic (noradrenergic or dopaminergic), glutamatergic and serotonergic (5-HT) neurotransmission have been observed in depressed patients [36]. Neuroendocrine dysregulation may also be a factor, with emphasis on three axes: hypothalamic–pituitary–adrenal and hypothalamic–pituitary–thyroid [37]. A molecular imbalance, characterized by increased levels of oxidative stress and low antioxidant status, has also been observed in patients with depression [38]. This would favor the appearance of immune responses and a pro-inflammatory environment, thus contributing to the pathology of depression [39]. Recently the relation between alterations in neuroplasticity and depression has received considerable attention. The term refers to the ability of the neural system to adapt to the internal and external stimuli and to respond adaptively to future stimuli. Neuroplasticity is of key significance in the brain’s adaptation to stress, which may underlie various psychiatric disorders, such as depression, post-traumatic stress disorder, etc., and is the basis of the so-called neuroplasticity theory, which suggests a decrease in neuroplasticity in the hippocampus and prefrontal cortex in depressed patients, as well as a decrease in the concentration of neurotrophic factors, such as brain-derived neurotrophic factor (BDNF), in subjects with depression. According to this theory, antidepressants would elevate the concentration of neurotrophic factors and improve neuroplasticity in the hippocampus and PFC [40].

Kell and colleagues found positive effects in subjects self-reporting low mood but not diagnosed with depression or another mood disorder and who were otherwise healthy [33]. Additionally, there is also growing evidence supporting the antidepressant and anxiolytic effects of saffron in humans suffering from depression and anxiety. Thus, saffron extracts can relieve the severity of symptoms of depression and the effect of saffron extracts resemble those of tricyclic (TCA), Selective Serotonin Reuptake Inhibitors (SSRI) and Selective Noradrenaline Reuptake Inhibitors antidepressants in depressed patients [41,42]. Saffron extracts, when administered in combination with pharmacological antidepressants, were also shown to improve some scores related to depression, even in subjects who had been using the antidepressants with no improvement [43]. In this case, the affron^®^ dose used was 14 mg b.i.d.

The active principles contained in saffron extracts, which account for the active antidepressants, are basically safranal and crocin isomers [44,45,46]. However, there is a wide variety of presentations that do not control the content of these bioactive molecules, making it very difficult to compare commercial brands in terms of pharmacological effectiveness. The safety and efficacy of safranal and crocin bioactive components have been described elsewhere, showing an exceptionally low toxicity, with an LD50 for normal cells of 20.7 g/kg [47]. In living animals, doses of the ethanolic extract up to 5 g/kg did not produce any demonstrable acute toxic effects in mice, and thus saffron is considered to have practically no acute toxic effect [48]. In our experiment, affron^®^, a standardized extract from Spanish *C. sativus* stigmas, was used.

Although there are a number of favorable clinical studies regarding the effects of affron^®^ on the modulation of the symptoms of depression and anxiety in humans [43,49,50,51,52,53,54,55,56,57,58,59], it is necessary to observe its acute and chronic effects in more controlled animal studies to further explore and understand the mechanisms of action of affron^®^ on mood, its safety and the role that the route of administration plays on those effects. The present study is intended to address this scientific need.

## 2. Results and Discussion

### 2.1. Effects of Affron^®^ in the Elevated Plus Maze Test

**Figure 1:**
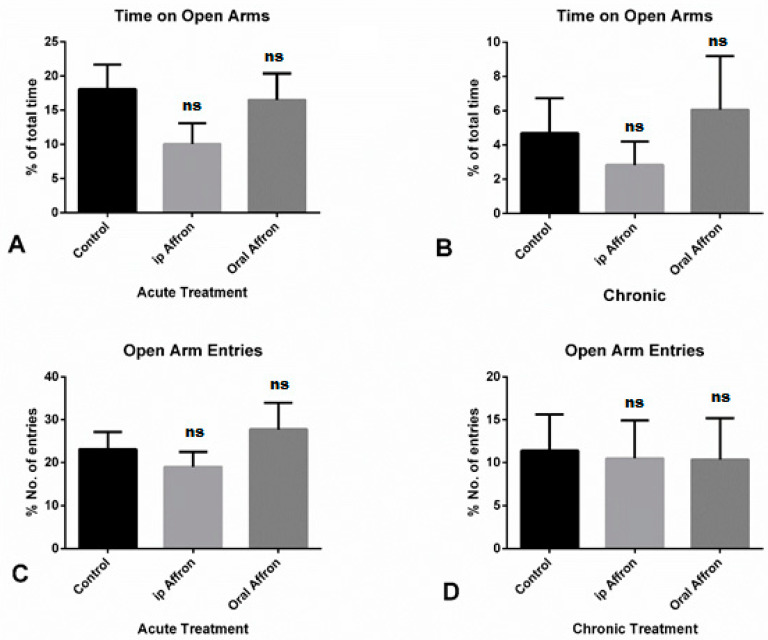
Effects of affron in the Elevated Plus Maze. (**A**,**B**): Percentage of time spent in open arms over the total in the acute and chronic treatment groups, respectively. (**C**,**D**): Percentage of entries in the open arms in the acute and chronic treatment, respectively. No significant effects were found between any of the groups in any condition (ns). The tests were administered on day one of treatment and on day 21 for the chronic group, 30 min after drug administration.

Affron^®^ was shown to be equally as ineffective either orally administered or by the intraperitoneal route. A one-way ANOVA showed that neither dose of affron^®^ (200 mg/kg p.o. and 50 mg/kg ip) produced changes in any of the anxiety-related parameters (time spent in the open arms and number of entries into open arms of the EPM) (*p* > 0.05, not significant (ns)). Bonferroni’s post hoc test confirmed that no statistically significant differences existed between treated and placebo groups (*p* > 0.05, ns) in acute and chronic treatments. No differences were observed for the time in open arms variable in any of the groups, as shown in Figure 1C,D. Values represent the mean ± SEM (*n* = 10 animals per group).

### 2.2. Effects of Affron^®^ in the Forced Swimming Test

**Figure 2:**
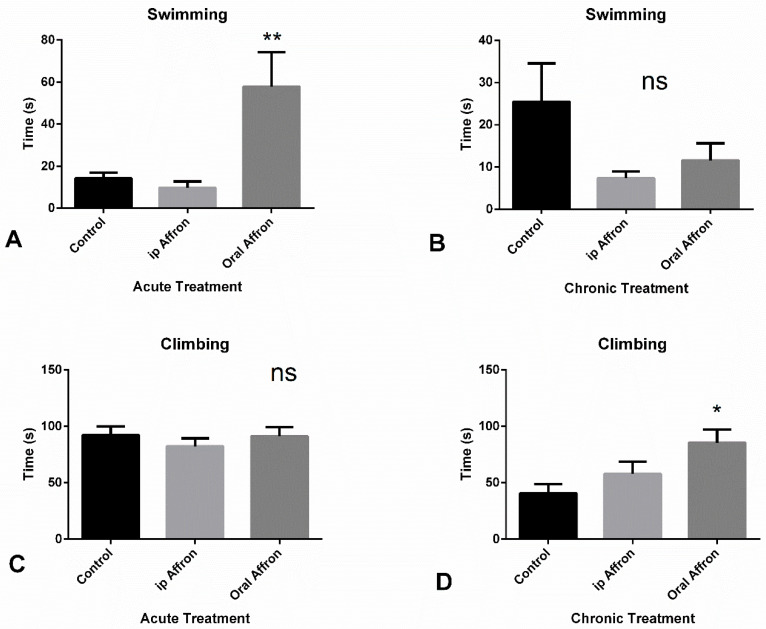
(**A**,**B**): Average time spent in immobility or swimming in the Porsolt test in animals receiving either an acute or a chronic treatment with affron through the intraperitoneal (gray column) or the oral (black column) route, respectively (** *p* < 0.01). (**C**,**D**): Average time climbing in the acute and chronic treatments, respectively (* *p* < 0.05; ns = Non Significant). The tests were administered on day one of treatment and on day 21 for the chronic group, 40 min after drug administration.

A one-way ANOVA revealed that affron^®^ (200 mg/kg, oral) administered 30 min before the FST significantly increased swimming time [F (2.27) = 7.37, *p* < 0.01], as shown in Figure 2A] but had no impact on climbing time or immobility time (ns) (data not shown). The post-hoc Bonferroni’s test showed that affron^®^, given at a dose of 200 mg/kg significantly increased the time of swimming in the oral group compared to the control and ip groups (*p* < 0.05 and *p* < 0.01, respectively). Concerning the antidepressant-like activity of affron^®^, after 20 days of treatment, an increase in climbing time was observed in the oral group [F (2,27) = 4.91, *p* < 0.05], as shown in Figure 2D. A one-way ANOVA did not find any statistically significant difference in the rest of the variables (ns); however, affron^®^ treatment, both oral and intraperitoneal, tended to decrease immobility time compared to the control group, which led to a possible antidepressant effect of this compound, as shown in Figure 2D. The post hoc test revealed a significant increase in climbing time in the oral group compared with the control group (*p* < 0.05), but there were no significant differences in the remaining groups (ns).

### 2.3. Effects of affron^®^ in the Sucrose Preference Test

**Figure 3:**
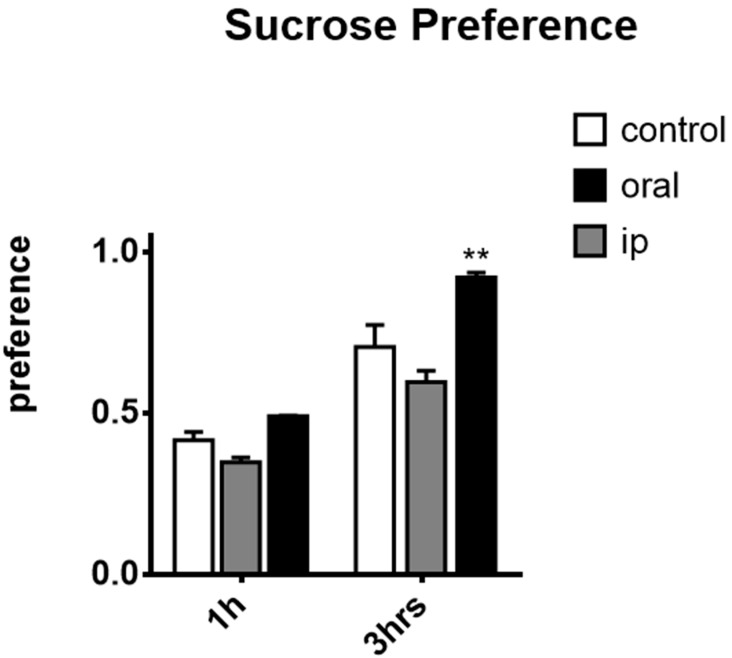
Sucrose preference test. Sucrose preference was calculated as the quantity of sucrose solution drunk/total fluid intake and is considered an index of the motivational state of the animal, (*n* = 10 per group) represented as mean ± SEM. Repeated-measures two-way ANOVA with Bonferroni post-hoc test: ** *p* < 0.001; differences between the oral group and the control group. The tests were administered on day one of treatment and on day 21 for the chronic group, 50 min after drug administration.

Repeated-measures two-way ANOVA found an overall interaction between time and treatments [F (6,78) = 12.30, *p* < 0.0001] and main effects of time [F (3,78) = 318.7, *p* < 0.0001] and treatment [F (2,26) = 19.20, *p* < 0.0001]. Subsequent analysis revealed that, as reflected in Figure 3, after an hour, a significant increase in the sucrose preference was found in the oral group with respect to the intraperitoneal group (*p* < 0.05), but there were no differences between the oral and intraperitoneal groups compared to the control group (ns). Statistically significant differences were observed in the 3 h measure in sucrose consumption between the oral group with respect to the control and intraperitoneal groups (*p* < 0.0001), but there was no difference between the control and the intraperitoneal groups (ns).

The results showed that the oral administration of affron^®^ may ameliorate some depressive-like behaviors both acutely and after long-term treatment. Interestingly, no effects were observed with the intraperitoneal administration. Additionally, repeated oral administration reduced anhedonic behavior, assessed in the sucrose preference test—an effect that was not observed under ip administration. Overall, these results are congruent with reports describing the beneficial effects of saffron extract consumption in patients suffering from anxiety or depression [41,42].

Considering the fact that drugs used to treat mental disorders are best studied in models of the disease, we used a rat model of depression, based on isolation [60]. Thus, individual housing has been shown to induce depressive-like behavior [61]. For that, animals were individually housed from arrival at the facilities until the end of the experiments, which allowed us to monitor any possible improvement or amelioration in behaviors related to anxiety, depression or anhedonia after affron^®^ supplementation.

Regarding the anxiolytic effects in the elevated plus maze, no significant differences were found between any of the groups in our study. In other studies, higher doses of saffron ip (56 and 80 mg/kg) showed anxiolytic effects in the EPM with a single dose, and a dose-dependent effect that decreased with doses up to 300 mg/kg [62]. It is possible that the lower dose used in our study limited the expression of the anxiolytic potential of affron^®^. It is also possible that differences in the source of plant material could explain the different findings in our study.

Data on the forced-swimming test assessing depressive-like behavior were clearer. On the one hand, affron^®^ did not affect immobility time or latency to the immobility period. However, a clear effect in the escape behavior (swimming and climbing) with the oral administration of affron^®^ can be observed. Our data are interpreted in line with the presence of antidepressant effect, since the animals clearly show more motivation to fight against the adverse conditions of being exposed to the water environment. These data appear relevant as they highlight the ability of saffron extracts to acutely improve depressive-like behavior.

To our knowledge, the antianhedonic effects of affron^®^ in the sucrose preference test have not been previously reported in animal studies. This is the clearest effect found with affron^®^ in the study. Oral dose was able to increase the preference for the sweet drink, which is indicative of a positive motivational state. Again, in this situation, oral administration was effective instead of intraperitoneal injection. Interestingly, intraperitoneal administration may even induce the opposite effect (i.e., anhedonia, indicative of desensitization of the brain reward system). It is worth mentioning that the antianhedonic response, measured by sucrose preference, differs in response to antidepressants with different mechanisms of action, such as SSRI and TCA [63].

The fact that the oral administration of affron^®^ was the effective route to observe the positive effects of this compound, and not ip administration, may be related to the fact that the active form of crocin isomers from saffron extract is crocetin, which is formed in the intestinal tract by glycosidases of enterocytes [64,65,66]. Thus, the saffron extract must, apparently, be taken orally to induce its antidepressant effect. In any case, oral administration is the preferred route for any potential nutraceutical since the majority of nutraceuticals are intended for this route of administration.

The neurobiological mechanisms that explain affron‘s antianhedonic effect and possible antidepressant effect have not been directly assessed yet. Regulation of mood was initially attributed to downregulations in monoamines, such as dopamine, serotonin or noradrenaline [67,68,69]. Whereas anhedonia is associated with low levels of dopamine, anxiety and depression appear to be more associated with the decreased activity of both serotonin and dopamine. The clear actions of affron^®^ in the amelioration of anhedonia suggest a more potent action in the regulation of dopamine than serotonin or other monoamines. Indeed, regarding dopamine modulation, it has been proven that saffron affected monoamine oxidases MAO-A and MAO-B in the brain [68], and the administration of *C. sativus* and its constituents increased brain dopamine levels in a dose-dependent manner [68]. These effects of *C. Sativus* in dopamine, together with the modulation of the excitatory amino acid, glutamate, and interactions with the opioid system have been reported to reduce withdrawal syndrome and may contribute to the amelioration of behavioral symptoms observed here [68].

However, the regulation of mood has been more recently associated with other factors, such as the alteration of neurotrophic factors, dysregulation in the hypothalamus–hypophysis–adrenal axis (HPA), low-grade inflammation or increased oxidative stress [70,71,72]. Certain evidence suggests that saffron regulates some of these mechanisms [73]. Indeed, saffron and its constituents, crocin and safranal, as well as crocetin, are potent antioxidants that can reduce oxidative stress, as demonstrated in animal models [74,75,76]. Its anti-inflammatory properties [77,78] and the modulation of the activity HPA in animal models of stress (i.e., by reducing levels of plasma corticosterone [79,80]) have also been proven. Future studies are needed to explore the specific actions of different doses of affron^®^ in these processes.

## 3. Material and Methods

### 3.1. Materials

Affron^®^ is a patented compound (ES2573542A1) and has been previously characterized [33]. Samples of saffron stigma extracts marketed under the brand name affron^®^ were provided by Pharmactive Biotech Products SL, standardized to ≥3.5% Lepticrosalides^®^, a term which characterizes the sum of the bioactive compounds safranal and crocin isomers, analyzed by HPLC [33]. The compound was presented in powder form and stored in darkness until the experiment was performed.

### 3.2. Animals

A total of 30 adult male Wistar rats (ENVIGO, Barcelona, Spain) weighting 300–350 g at the beginning of the experiments were kept under a 12-h light/dark cycle (lights off at 12:00 p.m.) in conditions of constant temperature (23 ± 1 °C). Standard food and tap water were available ad libitum at the home-cage outside the schedules assigned to experimental manipulation.

All experimental protocols adhered to the guidelines of the Animal Welfare Committee of the Universidad Complutense of Madrid, following European legislation (European Directive 2010/63/UE).

### 3.3. Experimental Design

**Figure 4:**
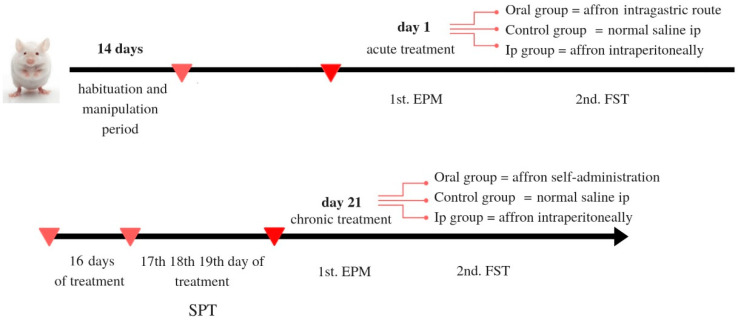
Schematic representation of the treatment schedule.

Animals had an acclimation period of one week, after which they were habituated to manipulation for several days before the experiment started. Upon arrival at the facilities, animals were housed in isolation for the duration of the study, according to a social isolation model of anxious/depressive-like behavior [81]. Animals’ weights were recorded every two days during the experiment. Rats were randomly assigned to one of the three of the following experimental groups: Oral affron^®^ (Oral), Intraperiotoneally administered affron^®^ (ip) and Vehicle (10 rats per group). In the Oral group, affron^®^ was dissolved in their tap water and the rats were monitored to ensure they consumed an adequate dose during the first hours of the morning (See Figure 4 for a schematic representation of the experimental schedule used).

Behavioral tests were performed on the first day of the experiment in order to assess the acute effects of the treatment, and also after the chronic treatment. The tests used were the elevated plus maze (EPM), the Forced swim test (FST) and the sucrose preference test (SPT).

#### 3.3.1. Treatment

The animals received a single dose of affron^®,^ dissolved in distilled water at the beginning of the experiment. Rats in the oral group received 200 mg/kg affron^®^, which was delivered via the use of an intra-gastric cannula in a volume of 2 mL/kg. The control group was treated with 0.9% saline solution via intraperitoneal injection (ip) in a volume of 2 mL/kg. The ip group received 50 mg/kg affron^®^ dissolved in saline. Thirty minutes after each administration, the animals were tested on the elevated plus maze (EPM) and 30 min later they were assessed in the forced swim test (FST). For the next 20 days, rats in the oral group had free access to 200 mg/kg affron dissolved in their drinking water until they consumed the whole daily dose, which took place in less than 4 h, after which tap water was reintroduced. The rest of the animals received their daily dose via ip. The animals underwent the sucrose preference test on the 17th day of the experiment 30 min after affron^®^ administration. Likewise, on the 21st day of the experiment, the animals were assessed at the elevated plus maze, and 30 min later, at the forced swimming test. All treatments were prepared and administered daily, and all tests took place between 9:00 and 14:00.

#### 3.3.2. Anxiety and Depression-Like Behavior

Several different models were used for the assessment of anxiety- and depression-related behaviors in rodents [82]. The Forced Swim Test and the Sucrose Preference Test were employed to assess any depressive-like behavioral responses, being some of the most commonly used tests to assess this kind of behavior in animal models [83,84]. The elevated plus maze test was chosen to assess anxiety-related behavior. In addition, in the chronic study, sucrose preference was used as a complementary test for anhedonia [63,85]. Anhedonia is defined as the inability to experience pleasure from rewarding or enjoyable activities and constitutes a core symptom of depression in humans [86]. The order of the tests was chosen to minimize any possible interference. Considering the relative distance and difference in environments between the animal facilities and the testing room, 10 min of acclimation were granted prior to the behavioral tests.

#### 3.3.3. Elevated Plus Maze Test

The elevated plus maze (EPM) is based upon the conflict between an innate aversion to open spaces and a tendency to explore new environments (Suo et al., 2013). The apparatus consisted of two open arms (50 × 10 cm), two closed arms (50 × 10 × 20 cm) and a central platform (10 × 10 cm), which raised to a height of 50 cm. The maze was placed in the center of a quiet room and testing was performed under dim light. Each animal was gently placed on the central platform facing one of the closed arms and allowed to explore the maze for 5 min. The light of the test room was adjusted at 350 lux at the center of the maze. Then, the animals were removed and the EPM was cleaned with 30% ethanol between each test to prevent interference resulting from any residual odors of the previous rat. Data were registered automatically using the Mazesoft software. The analysis of exploratory activity in rats included four parameters: number of entries into open arms; number of entries into closed arms; time spent in open arms; time spent in closed arms.

#### 3.3.4. Forced Swim Test (Porsolt Test)

The forced swimming test (FST) was originally introduced in 1977 by Porsolt and has since become a standard for the evaluation of antidepressant drugs [87]. In preparation, 24 h prior to the test, animals were placed in the testing apparatus for 10 min in order to become familiar with the testing environment and to minimize novelty effects [82,88]. Briefly, the rats were individually placed in a transparent methacrylate cylinder (height 50 cm, diameter 30 cm) filled with water (23 ± 1 °C) to a height of 40 cm (a modified version of the FST was used to increase water depth to 40 cm, so the rats were unable to touch the bottom of the tank). Water was replaced for each test. Following each test session, rats were dried using cotton towels and returned to their home cages. All sessions were recorded with a video camera (SONY HDR-CX115, New York, NY, USA). Afterwards, four behavioral categories were quantified using the freeware for behavioral quantification, Raton Time 1.0 (Fixma SL, Valencia, Spain), including immobility latency (latency to immobility), immobility (rat floating in the water with only movements necessary to keep the nose above water), swimming (active horizontal movements around the cylinder) and climbing (upward-directed movement of the forepaws, usually directed against the walls). Animals underwent the test 30 min after the elevated plus maze.

#### 3.3.5. Sucrose Preference Test

The sucrose preference test (SPT) is a reward-related test commonly used as an indicator of anhedonia [89]. No previous food or water deprivation was applied before the test. During the adaptation period, the animals were presented in their home cages with two bottles of the type used for the test, in order to habituate them to testing conditions. The rats were allowed simultaneous access to two identical drinking bottles that contained a 1% sucrose solution for 3 h. The sucrose solution was prepared daily before the experiment and kept at 4 °C for no longer than 24 h. The position of the drinking bottles was changed after each measurement to exclude the effect of place preference [89,90]. The consumption of water and sucrose solution was calculated by weighing each bottle before, during and after the test. Sucrose preference was calculated as the quantity of sucrose solution drunk/total fluid intake [2]. The sucrose preference test was performed on the 17th day of treatment in order to avoid interferences with the other tests.

### 3.4. Statistical Analyses

The data are expressed as mean ± standard error of mean (SEM). Normality data were assessed by D’Agostino and Pearson test. Data were analyzed using GraphPad Prism version 6.0. In order to detect significant differences among the experimental groups, depending on the behavioral test, either one-way analysis of variance with one factor (treatment) or two-way ANOVA comparing two factors—treatment (oral, intraperitoneal, control) and time—was used. Data of the behavioral tests were analyzed as dependent variables, and the Bonferroni post-hoc test for multiple comparisons was used when appropriate. Values of *p* ≤ 0.05 were considered statistically significant.

## 4. Conclusions

In conclusion, this study provides evidence of the antianhedonic, and mild antidepressant actions of a 50 mg/kg acute ip dose and a 200 mg/kg oral dose of affron^®^, a standardized saffron extract, when administered acutely or repeatedly, orally. Future studies are required to ascertain the specific mechanisms of this action. Anyhow, these results open new fields for the possible application of affron^®^ to prevent negative emotional states or as a co-adjuvant therapy in the treatment of depression.
